# Role of inflammation in depression relapse

**DOI:** 10.1186/s12974-019-1475-7

**Published:** 2019-04-17

**Authors:** Chun-Hong Liu, Guang-Zhong Zhang, Bin Li, Meng Li, Marie Woelfer, Martin Walter, Lihong Wang

**Affiliations:** 1 Beijing Hospital of Traditional Chinese Medicine, Capital Medical University, Beijing Institute of Traditional Chinese Medicine, Beijing Key Laboratory of Acupuncture Neuromodulation, Beijing, 100010 China; 2 Dermatological Department, Beijing Hospital of Traditional Chinese Medicine, Capital Medical University, Beijing, 100010 China; 3 Acupuncture and Moxibustion Department, Beijing Hospital of Traditional Chinese Medicine, Capital Medical University, Beijing, 100010 China; 4 Clinical Affective Neuroimaging Laboratory (CANLAB), Otto-von-Guericke-University Magdeburg, Magdeburg, 39120 Germany; 5 Department of Biomedical Engineering, New Jersey Institute of Technology, Newark, NJ 07102 USA; 6 Department of Psychiatry and Psychotherapy, University of Tuebingen, Tubeingen, 72074 Germany; 7 Leibniz Institute for Neurobiology, Magdeburg, 39118 Germany; 8 Department of Psychiatry, University of Connecticut Health Center, Farmington, CT 06030 USA

**Keywords:** (Neuro) inflammation, Recurrent major depressive disorder, Functional magnetic resonance imaging, Vagus nerve stimulation

## Abstract

Major depressive disorder (MDD) is a leading cause of disability worldwide. After the first episode, patients with remitted MDD have a 60% chance of experiencing a second episode. Consideration of therapy continuation should be viewed in terms of the balance between the adverse effects of medication and the need to prevent a possible relapse. Relapse during the early stages of MDD could be prevented more efficiently by conducting individual risk assessments and providing justification for continuing therapy. Our previous work established the neuroimaging markers of relapse by comparing patients with recurrent major depressive disorder (rMDD) in depressive and remitted states. However, it is not known which of these markers are trait markers that present before initial relapse and, consequently, predict disease course. Here, we first describe how inflammation can be translated to subtype-specific clinical features and suggest how this could be used to facilitate clinical diagnosis and treatment. Next, we address the central and peripheral functional state of the immune system in patients with MDD. In addition, we emphasize the important link between the number of depressive episodes and rMDD and use neuroimaging to propose a model for the latter. Last, we address how inflammation can affect brain circuits, providing a possible mechanism for rMDD. Our review suggests a link between inflammatory processes and brain region/circuits in rMDD.

## Introduction

**Fig. 1:**
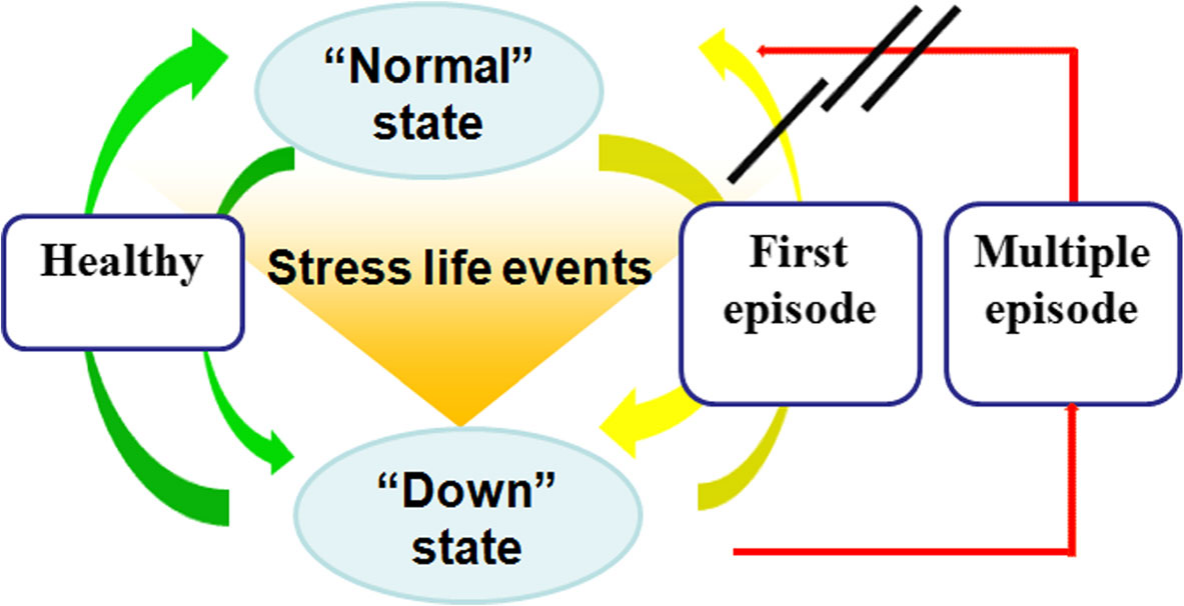
Neural dysfunction becomes increasingly serious in rMDD with increased numbers of depressive episodes. In health, the tendency to enter a “down state” is relatively low, and the ability to return to a “normal state” is relatively rapid and complete (green arrows). In individuals with first-episode MDD, the tendency to enter a down state is relatively high, and the ability to return to a normal state is impaired slightly (yellow arrows). This ability becomes impaired more seriously in individuals with rMDD (red arrow). The down state itself is not abnormal in this model

Major depressive disorder (MDD) is characterized by impaired mood, anhedonia, ruminative thoughts, impaired cognition, and impaired attentional control [1]. These features significantly affect patients’ occupation and life and increase the burden on their families and society [2, 3]. Furthermore, patients with MDD can experience a chronic deteriorating disease course. Indeed, 34–83% of MDD patients develop a new depressive episode within 6 months [4], and on average, MDD patients experience about five depressive episodes throughout their lifetime [5, 6]. Notably, the number of previous depressive episodes is associated with the possibility of recurrence [7, 8]. Sixty percent of patients with remitted MDD carry a risk of developing a new depressive episode after the first episode, 70% after the second, and 90% after the third episode [9, 10]. Furthermore, normal neuronal function is more difficult to achieve in patients with recurrent MDD (rMDD) with a higher number of depressive episodes (Fig. 1).

The heterogeneous nature of depression requires objective characterization, including in assessment of depression severity and treatment response, for classification of MDD subtype [11]. However, clinical features alone are not sufficient to guide precise decision-making regarding medications or prevention of relapse. Therefore, biomarkers are needed to facilitate characterization of rMDD subtypes and decisions on effective individualized therapy. Here, rMDD is defined based on 17-item Hamilton Depression Rating Scale (HAMD) scores. HAMD ≤ 7 for at least 2 weeks was considered to indicate an rMDD state [12]. Clarifying the effects of rMDD on the brain could be a crucial step towards early differential diagnoses and effective therapy.

In this review, we discuss the potential role of inflammation and immunological mechanisms that might play an essential part in depression relapse. First, we outline how inflammation may translate to subtype-specific clinical features and suggest how this could be used for diagnostic and treatment purposes. Next, we emphasize the relationship between pro-inflammatory cytokines and the brain in MDD. Third, we present an important link between the number of depressive episodes and rMDD and propose a model for the latter. Fourth, we address how inflammation can affect brain circuits, thereby providing a possible mechanism for rMDD.

## Link between inflammation and rMDD

Recent evidence concerning the epidemiology, symptoms, and complications of MDD have been documented. Nevertheless, the biological mechanisms that underpin MDD are incompletely understood [12]. Historically, the “monoamine-depletion hypothesis” has been the main viewpoint; in this hypothesis, an imbalance, mainly between serotonergic and noradrenergic neurotransmission, is proposed to be the basis of MDD pathophysiology [13, 14]. However, the monoamine-depletion hypothesis alone cannot fully explain the pathogenesis of depression [14].

Over the past two decades, research has suggested that inflammatory processes are involved in the onset and maintenance of MDD, and the “inflammatory hypothesis” has been proposed [15]. Notably, chronic inflammation may contribute to serotonergic, noradrenergic, and dopaminergic dysfunction [16, 17]. Depression relapse and lack of therapeutic benefits of antidepressants might be associated with overall activation of the inflammatory response [18]. Therefore, immune dysregulation or chronic inflammation might be present in rMDD [19]. The evidence underlying this hypothesis is discussed in more detail below.

In recent years, the association between inflammation and MDD has been investigated with growing interest [20]. Extant data suggest that patients with MDD display eight main abnormalities (Table 1). The first abnormality is increased levels of pro-inflammatory cytokines such as tumor necrosis factor-α and interleukin (IL)-6 and of C-reactive protein (CRP), as well as decreased levels of circulating cytokines such as IL-1β and IL-8, in the blood and cerebrospinal fluid of patients with rMDD [21]. The second abnormality is that increased levels of pro-inflammatory cytokines might lead to increased activity of corticotropin-releasing hormone and hyperactivity of the hypothalamic−pituitary−adrenal (HPA) axis, which has been described broadly in MDD progression and is potentially useful for predicting recurrence [15]. Third, higher levels of IL-6 or CRP can predict subsequent development of depressive symptoms (e.g., tiredness, lack of energy, sleep problems, and changes in appetite) [23, 24]. Fourth, increased inflammatory activity leads to greater susceptibility to treatment-resistant depression (TRD) [25]. Fifth, intestinal microbes can influence tryptophan metabolism and cause changes in levels of gamma-aminobutyric acid, dopamine, and serotonin [26, 27]. Sixth, cognitive behavioral therapy may influence inflammatory processes and be associated with a reduction in inflammation over time [28]. Seventh, vagus nerve stimulation (VNS) can relieve MDD symptoms by modulating the inflammatory response [29, 30]. Cervical VNS was approved by the US Food and Drug Administration for managing chronic treatment-resistant MDD in 2005 [30]. Finally, inflammation can elicit symptoms of anhedonia and motor retardation [31, 32].

**Table 1:** Summary of the eight main abnormalities related to inflammation and associated consequences in patients with MDD

Main abnormalities	Consequence
Increased levels of TNF-α, IL-6, and CRP and decreased levels of IL-1β and IL-8 [22]	rMDD
Increased activity of corticotropin-releasing hormone and hyperactivity of the HPA axis [15]	MDD progression and predicted recurrence
Higher levels of IL-6 or CRP [23, 24]	Subsequent development of depressive symptoms (e.g., tiredness, lack of energy, sleep problems, and changes in appetite)
Increased inflammatory activity [25]	TRD
Intestinal microbes [26, 27]	Tryptophan metabolism and the levels of gamma-aminobutyric acid, dopamine, and serotonin
Cognitive behavior therapy [28] Vagus nerve stimulation [29, 30] Inflammation [31, 32]	Inflammatory processes Relief of MDD symptoms Anhedonia and motor retardation

Abbreviations: *CRP* C-reactive protein, *HPA* hypothalamic−pituitary−adrenal, *IL-6* interleukin-6, *rMDD* recurrent major depressive disorder, *TNF-α* tumor necrosis factor-α, *TRD* treatment-resistant depression

Based on the findings described above, we would argue that the inflammation derives from a MDD subgroup. Moreover, further evidence has demonstrated a direct link between inflammatory processes and rMDD [17]. For instance, Freeman et al. demonstrated that CRP levels were associated with depression relapse (odds ratio = 1.92; 95% confidence interval 1.43–2.55; *p* < 0.0001), suggesting that inflammation contributes to the risk of relapse [33]. Copeland et al. also demonstrated that cumulative episodes predict changes in CRP levels independently, although body mass index, nicotine use, and recent infections were partial mediators of the effect of cumulative episodes on CRP levels [34]. Several clinical trials involving innovative neuroimmune interventions are underway and will hopefully have an impact on future clinical care [35]. Indeed, exciting advances in psychoneuroimmunology have raised hopes of improving the prediction and treatment of depression relapse.

## Relationship between pro-inflammatory cytokines and the brain in MDD

Communication between the periphery cytokines and MDD was first detected in the early 1990s [36], leading to the formulation of the inflammatory hypothesis of depression [37]. Experimental and clinical evidence suggests that the abnormal profile of pro-inflammatory cytokines, especially IL-1, IL-6, IL-1β, tumor necrosis factor alpha (TNF-α), and of the acute phase reactant CRP may contribute to the initiation, relapse, and progression of MDD [38–40]. It is important to mention, though, that the levels of these cytokines are increased to a much lesser extent than in autoimmune or infectious diseases [41, 42]. Moreover, elevated CRP and IL-6 were found significantly more frequently in individuals with rMDD than in healthy controls [42, 43], and depression was associated with higher CRP levels, especially in the case of cumulative episodes of depression [34]. Similarly, higher baseline CRP and IL-6 levels are not only associated with cognitive symptoms of depression but also predict cognitive symptoms at an average follow-up of 11.8 years, suggesting that inflammation precedes the progression of MDD [44]. It is noteworthy that peripheral cytokines aid inflammatory processes and the immune system to form coordinated responses to infection, which are produced by a broad range of cells including macrophages, B lymphocytes, T lymphocytes and mast cells, endothelial cells, fibroblasts, and various stromal cells [45]. Communication between the periphery and the brain has been postulated to occur as (1) a peripheral immune effect or (2) a central immune effect, such as (a) an immune type of cell effect (activated microphages and micro-glial cells), (b) cytokine transfer across the blood–brain barrier, or (c) HPA interaction with stress and neurotransmitters.

A vast amount of data has demonstrated that increased inflammation and hyperactivity of the HPA axis are among the most consistent biological findings in subsets of MDD patients and are often associated with each other [46]. Bhagwagar et al. found increased salivary cortisol in remitted MDD patients at high risk for recurrence, indicating that HPA dysregulation may be a marker of an unfavorable disease course [47]. Chronic cytokine exposure may influence the HPA axis via glucocorticoid receptor function [48–50]. Specific cytokine signaling molecules (including p38 mitogen-activated protein kinase (MAPK), nuclear factor kappa-light-chain-enhancer of activated B cells (NF-κB), signal transducer and activator of transcription 1a, and cyclooxygenase-2 (COX-2)) have been shown to be involved in the disruption of glucocorticoid receptor activity [38]. HPA axis hyperactivity is a marker of glucocorticoid resistance and could lead to immune activation. Equally, cytokines can stimulate HPA axis activity via glucocorticoid receptor function at multiple levels [48]. In addition to the glucocorticoid receptor signaling pathways, cytokines have been found to affect the synthesis, reuptake, and release of monoamine neurotransmitters [51]. Several proinflammatory cytokines can induce the enzyme indoleamine 2,3 dioxygenase (IDO), which catabolizes tryptophan, the precursor of serotonin, into kynurenine [52]. In this way, the breakdown of tryptophan leads to reduced serotonin synthesis, which is directly linked to the etiology of depression [53]. By activating MAPK pathways, inflammatory cytokines increase the expression and function of the serotonin, norepinephrine, and dopamine reuptake pumps (transporters) [16]. Taken together, these cytokine-driven effects on neurotransmitter biochemistry may explain the observations that increased inflammation is associated with less robust antidepressant treatment responses in chronic forms of MDD [16] and that increased inflammatory markers may possibly underlie medical problems associated with rMDD [54, 55].

The peripheral immune changes and the central immune activation (e.g., macrophage accumulation and microglia activation) are in continual communication [56, 57]. Microglial cells are the brain’s immune cells [58]. Chronic activation of perivascular and parenchymal microglia expressing pro-inflammatory cytokines was associated with an increase in the brain’s production of reactive oxygen species, leading to greater susceptibility to neuronal damage and death [59]. Neuroimaging studies using ^18F^FEPPA positron emission tomography have noted microglial activation in the prefrontal cortex, anterior cingulate cortex (ACC), and insula in MDD, and translocator protein total distribution volume in the ACC was found to be correlated with depression severity [60]. Tong et al. found reductions in numbers of endogenous hippocampal microglia and in hippocampal microglial activation in mice, following treatment with different types of chronic stress [61]. Moreover, microglia produce IDO, which converts tryptophan to kynurenine [62]. The kynurenine pathway was proposed to serve as the switch from acute effects of inflammatory challenges and stress to the development of depression [63]. Collectively, these central and peripheral immune alterations lead to elevated pro-inflammatory cytokine production, which has been implicated in the development and recurrence of MDD [64, 65].

Epidemiologically, chronic immune dysregulation is associated with a higher incidence of mood disorders. For example, the incidence of both MDD and bipolar disorders increases relative to the general population after the diagnosis of immune-mediated disease, e.g., inflammatory bowel disease, multiple sclerosis, or rheumatoid arthritis [66]. Recent meta-analyses have found decreased levels of kynurenine in MDD versus healthy controls but not in bipolar disorder [67]. Using high-sensitivity testing of fasting plasma CRP levels at baseline and after treatment, Chang and colleagues reported that a baseline CRP level of 621.6 ng/mL could discriminate between bipolar disorder II and MDD [68]. However, Powell et al. found that reduced transcription of IL8 could be used to distinguish both MDD and bipolar disorder from control subjects [69]. This may suggest that both MDD and bipolar disorder patients have a common molecular pathway affecting the transcription of IL8 [69]. Although kynurenine and CRP could be used to differentiate between MDD and bipolar disorder patients, we could not determine whether these alterations would persist once the patients were in other mood states. Future research should aim to replicate findings detailed in this exploratory study in a larger medication-free sample and examine whether identified biomarkers could be used prospectively to aid clinical diagnosis.

## Neuroimaging biomarkers related to rMDD

Several brain regions have been implicated in MDD pathophysiology, but global efforts to identify sensitive, specific, and clinically predictive brain correlates of depression relapse have not succeeded. Using high-dimensional data clustering based on current symptoms of depression and anxiety, Maglanoc et al. found that the strongest differences between depression subgroups (based on symptom profiles) were in the connecting edges between a default mode network (DMN) component and the fronto-temporal network, and between the precuneus and fronto-temporal network [70]. Furthermore, Drysdale et al. recently demonstrated that clinical dimensions overlap, to a certain degree, with neurophysiological subtypes (“biotypes”) established based on the patient brain connectome, and that the brain connectome is able to predict treatment response in MDD [71]. Crucially, this work adds to accumulating evidence on the many biological markers, of which only a few are valid for the diagnosis and treatment of MDD in routine clinical practice [72]. Overcoming these hurdles will require identification of the specific subtypes of MDD (rather than seeking biomarkers that can identify *Diagnostic and Statistical Manual of Mental Disorders*-defined disorders), and it will require focusing on clinically meaningful differences between relevant clinical populations (rather than hypothesis-rejection versus healthy controls) [73]. Such MDD subtypes promise greater ecological validity and represent meaningful diagnostic targets for which is a great need in order to make optimal clinical decisions. At later stages, the number of previous episodes/relapses may be a useful criterion to identify rMDD patients. However, it would be of great value to identify patients with an increased risk of relapse before they enter the “path of recurrence”, as each additional episode will shape the neurobiological risk profile more adversely.

A vast body of research suggests that the number of previous depressive episodes has the strongest influence on MDD relapse, and that aberrant network topology (e.g., DMN and striatum aberrations) is the best predictor of depressive relapse [74]. A recent meta-analysis demonstrated that reductions in gray-matter volume in the dorsolateral prefrontal cortex (dlPFC) and dorsomedial prefrontal cortex (dmPFC) are evident in rMDD patients [75]. Using voxel-based morphometry, Serra-Blasco et al. revealed negative correlations between volume reduction of the right dmPFC and left insula and longer duration of illness in MDD patients at various stages of the illness (first episode, remitted–recurrent, and treatment-resistant/chronic MDD groups, with 22 patients in each group) [76]. Using vertex-based cortical thickness, Treadway et al. found that the number of previous episodes was associated with reduced volume in the dentate gyrus, and that cortical thinning of the left dmPFC was associated with a greater number of prior depressive episodes, but not with current depressive diagnosis [77]. Using voxel-based morphometry, Stratmann et al. found that the gray-matter volume in the right hippocampus and right amygdala was correlated negatively with the number of depressive episodes [78]. In addition to structural magnetic resonance imaging (MRI) changes, alterations in resting-state functional MRI (fMRI) have been observed in MDD. Using resting-state fMRI- and graph-based methods, Meng et al. demonstrated that aberrant topology of the right putamen network was correlated positively with the number of depressive episodes [79]. Using independent-component analyses, Greicius et al. found that the functional connectivity (FC) in the subgenual ACC (sgACC) was correlated positively with the duration of the current depressive episode [80]. Using the posterior cingulate cortex (PCC), amygdala, and sgACC as “seeds”, Jacobs et al. found that experiencing multiple episodes was associated with hypoconnectivity between the left PCC and multiple regions of cognitive control, and with hypoconnectivity between the amygdala and large portions of the salience network (SN) [81]. Despite a wealth of structural and functional neuroimaging studies on MDD, the findings have been heterogeneous, mainly owing to differences in data acquisition and processing, statistical analyses, and the clinical variables tested [82].

To understand the mechanisms underlying rMDD, MDD patients must be studied during remission and long-term prospective follow-ups must be conducted [83]. A prospective cohort study assessed 75 patients with remitted MDD who had not taken psychotropic medications and evaluated them during 14 months of clinical follow-up; 31 were found to be in stable remission and 25 developed a recurrent episode. Using a self-blame-selective task (e.g., “feeling guilty for everything”), Lythe et al. found, using fMRI, that the rMDD group exhibited higher connectivity between the right superior anterior temporal lobe and both the sgACC and adjacent septal region compared with the stable MDD group and healthy controls [84]. Another prospective cohort study investigated 47 medication-free remitted MDD patients for 14 months and showed that 30 patients remained resilient, whereas 17 experienced a recurrent episode. Using FC analyses with the left sgACC as a seed for resting-state fMRI, Workman et al. (2016) demonstrated that attenuated interhemispheric left-to-right sgACC connectivity could distinguish resilient patients from rMDD cases [85]. Irrespective of the inconsistent results, converging evidence suggests that MDD is consistently associated with DMN abnormalities, such as in the dmPFC and ACC. These dmPFC and ACC aberrations are associated with the number of depressive episodes and illness duration [77, 80]. Zaremba et al. (2018) recruited 64 MDD patients with a moderate or severe depressive episode at baseline, and subdivided them into groups of patients with and without relapse depending on the course of illness during follow-up. They found that MDD patients who had suffered a relapse showed a significant decline in insular and dlPFC volume [86]. Taken together, these findings on the DMN, insula, and dlPFC in patients with rMDD shed light on the development of a “bottom-up” processing bias regarding emotional stimuli (e.g., limbic area aberrations) and a disruption of top-down executive functions (e.g., dlPFC and DMN aberrations). The neuroimaging findings related to the number of depressive episodes, illness duration, and depression relapse are summarized in Table 2.

To date, MDD has been associated with aberrant brain activity and abnormal FC [87, 88]. A recent systematic review and meta-analysis showed that inflammatory biomarkers such as CRP and IL-6 had a small but significant association with the subsequent development of depressive symptoms in MDD [89]. Jokela et al. found that the level of CRP was positively correlated with a range of depression symptoms, particularly tiredness, lack of energy, sleep problems, and changes in appetite; the level of CRP was also associated with the depressive cognitive and emotional symptoms (e.g., anhedonia, depressed mood, reduced feelings of self-worth, poor concentration, and suicidal ideation) [23]. Stewart et al. found that greater depressive symptom severity at baseline was associated with larger 6-year increases in serum IL-6, as well as a weak bidirectional relationship between depressive symptom severity and CRP [90]. A systematic review and meta-analysis of 32 studies also supported an association of both CRP and IL-6 with depression in older adults, and it is likely that inflammation leads to depression [24]. Yet, neuroimaging findings and peripheral biomarker measurements in patients with rMDD are relatively scarce. A recent study protocol of a double-blind randomized placebo-controlled trial suggested that increased inflammatory activity indicated a greater susceptibility to TRD [25]. Future studies should thus investigate whether there is any cross-sectional or longitudinal association of peripheral biomarker measurements with neuroimaging in depression relapse.

## Proposed model of rMDD

Early diagnosis of patients based on intrinsic biomarkers (before the development of a recurrent biological effect on the brain) is reliant on neurobiologically rooted susceptibility rather than individual factors, for example, treatment history. Indeed, rMDD has been estimated to be associated with a greater familial risk than non-rMDD [91, 92]. Kruschwitz et al. provided corroborating evidence for a specific rMDD profile based on resting-state fMRI, which was itself found to be heritable and influenced by genetics [93]. In a cross-sectional, resting-state fMRI study, our research team reported that there was an increased fractional amplitude of low-frequency fluctuations (fALFF) in the left middle frontal gyrus in MDD patients and unaffected siblings compared with healthy controls, and that dysfunction in the left middle frontal gyrus may represent an imaging endophenotype, possibly indicating resilience rather than risk of MDD [94]. In a subsequent resting-state fMRI study by our research team, there was a lower fALFF in the right posterior insula and right precuneus and an increased fALFF in the left ventral anterior cingulate cortex (vACC) in rMDD patients in a depressive state compared with rMDD patients in a remitted state and healthy controls; lower fALFF in the right precuneus and increased fALFF in the right middle insula were correlated with the number of depressive episodes in all rMDD groups [95]. Taken together, these data led to a hypothesis that, although reduced fALFF in the right precuneus can be partially recovered, there is a greater reduction in the resting activity in the precuneus with an increased number of depressive episodes. MDD can be explained (at least in part) by the relationship between the DMN, central executive network (CEN), and SN [96, 97]. Hence, we proposed a model focusing on altered brain regional resting-state activity influencing neural network behavior, which predisposes individuals to depression relapse.

**Fig. 2:**
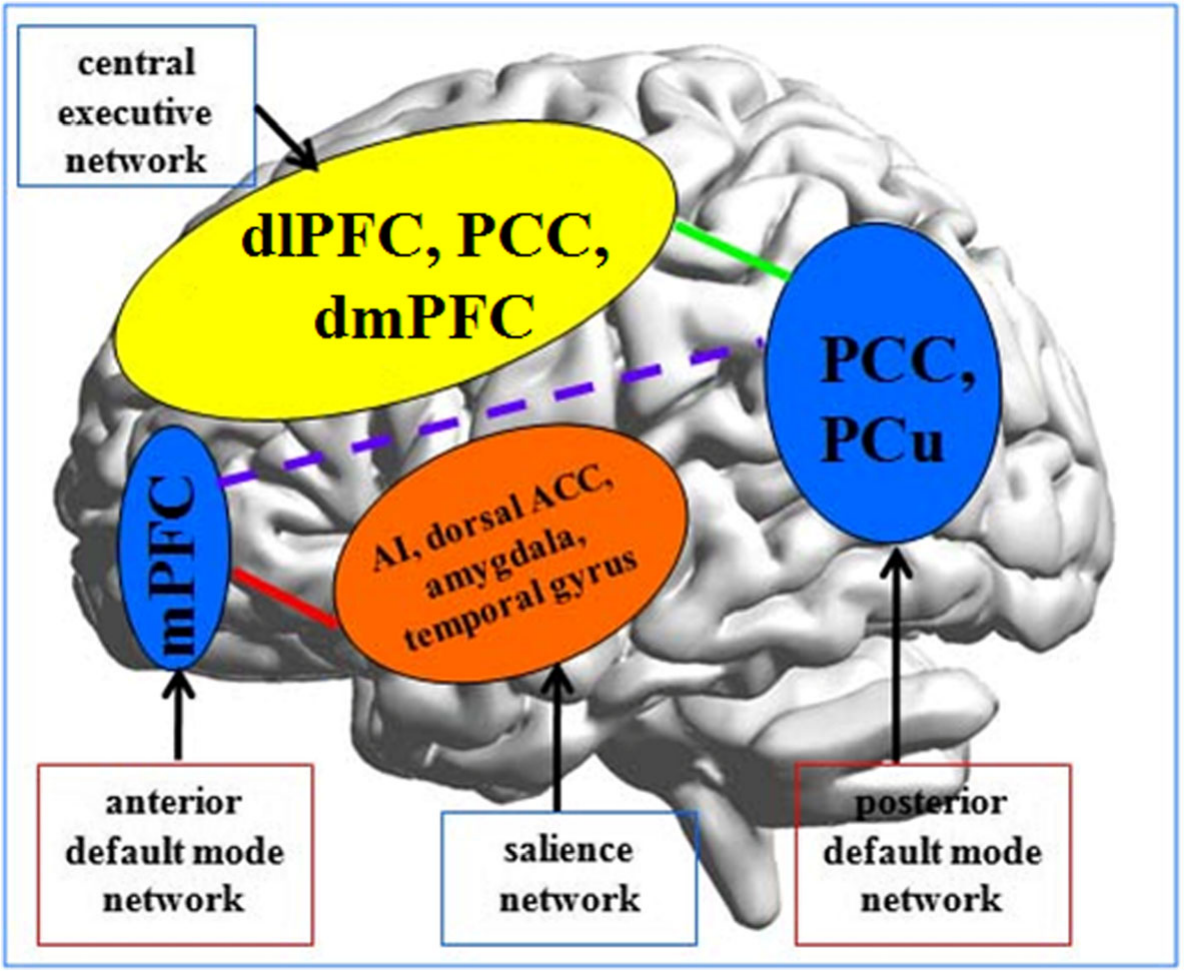
Proposed model of rMDD (schematic). Segregation of the anterior and posterior DMN could be an indicator of recurrent depression. Increased functional connectivity between the anterior DMN and the salience network may be an early sign of depression recurrence. Decreased functional connectivity between the posterior DMN and central executive networks is unique to rMDD. ACC, anterior cingulate cortex; AI, anterior insula; dlPFC, dorsolateral prefrontal cortex; dmPFC, dorsomedial prefrontal cortex; DMN, default mode network; mPFC, medial prefrontal cortex; PCC, posterior cingulate cortex; PCu, precuneus;; rMDD, recurrent major depressive disorder

The central role and relevance of the DMN in rMDD was outlined by Marchetti et al. [1]. The precuneus is the key brain region within the DMN, so we emphasize the important role of DMN disconnection in the pathophysiology of rMDD. Following the work of Mulders et al. [88], we can focus further on the separation of the anterior and posterior components of the DMN. Within the different disconnections of the DMN, increased FC between the anterior DMN and SN might be an early sign of depression relapse, but it is not specific to rMDD. However, decreased FC between the posterior DMN and CEN may be more specific to rMDD (Fig. 2).

## Association between brain regions, networks, and inflammation

**Fig. 3:**
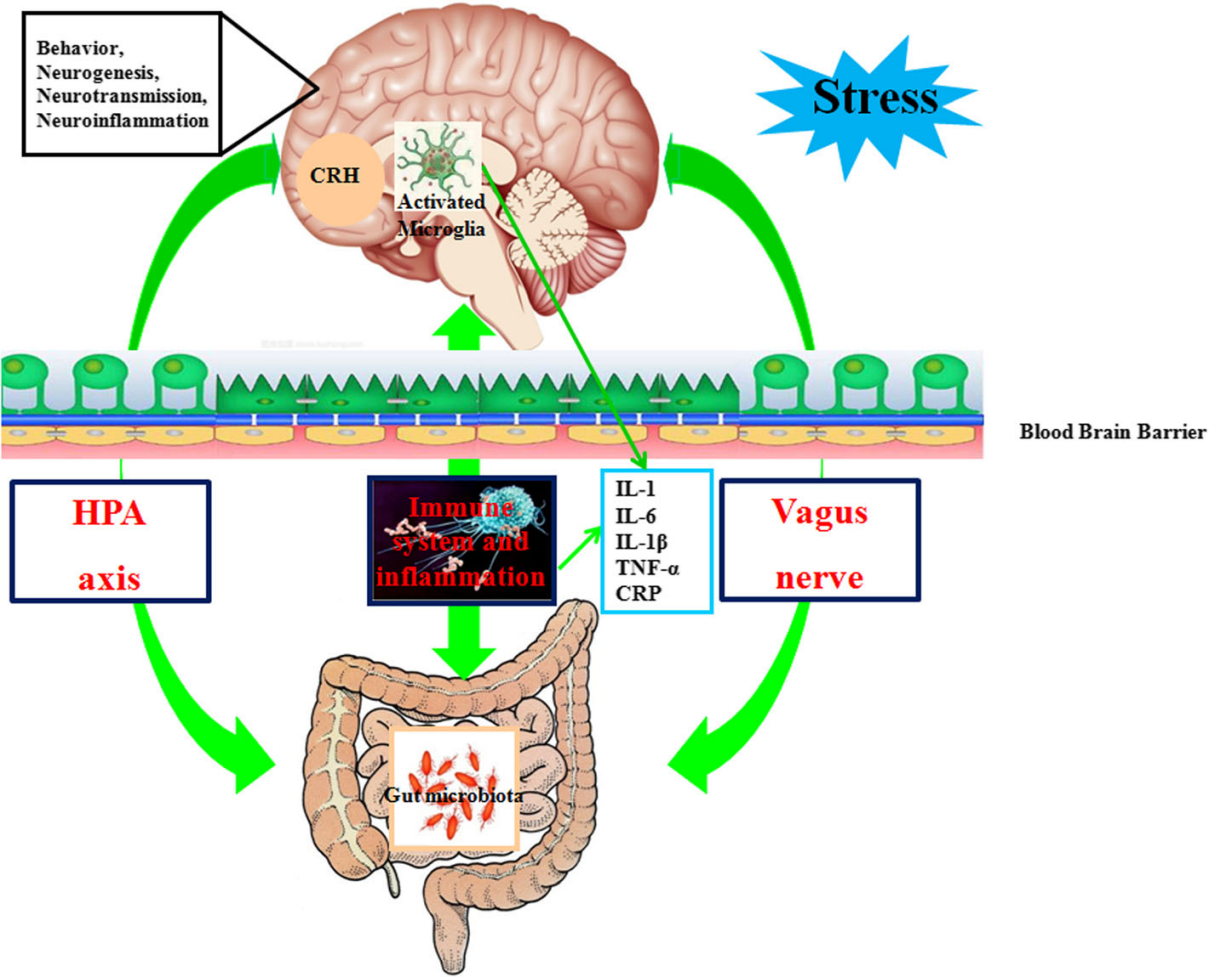
Relationship between inflammation and rMDD. Stress is an important factor in the occurrence of depression relapse. Neuroendocrine- and inflammation-related signals generated by gut microbiota and specialized cells within the gut can, in principle, affect the brain and may lead to release of neurotransmitters, excessive activation of microglial cells, increased levels of inflammatory factors from the peripheral nervous system and the central nervous system, release of inflammatory cytokines by immune macrophages, and depressive-like behaviors. *CRH* corticotropin-releasing hormone, *CRP* C-reactive protein, *HPA* hypothalamic–pituitary–adrenal, *IL* interleukin, *TNF-α* tumor necrosis factor-α

Neuroimaging data have revealed that a wide range of brain regions and networks may be involved in rMDD, which reflects relationships between the brain regions and their inflammation status. A negative correlation was found between plasma levels of CRP and decreased connectivity between the ventral striatum and ventromedial prefrontal cortex (vmPFC) in medically stable MDD; connectivity between the striatum and vmPFC was associated with increased plasma levels of IL-6, IL-1β, and IL-1 receptor antagonists in medically stable MDD [98]. MDD patients with a greater number of depressive episodes had a thinner medial prefrontal cortex (mPFC), and the CRP level was inversely associated with the thickness of the right mPFC [99]. Smaller hippocampal volumes and increased levels of the pro-inflammatory proteins IL-6 and CRP in MDD were also highlighted by Frodl et al. [100]. Structural or functional changes associated with inflammation are located mainly in the striatum, mPFC, and hippocampus in individuals with rMDD (Table 3). Depression relapse can also be due to activation of the HPA axis and vagus nerve via modulation of the immune system or production of bacterial metabolites, respectively (Fig. 3). The vagus nerve projects onto the nucleus tractus solitarius and connects further with the locus coeruleus, parabrachial nucleus, thalamus, hippocampus, and mPFC, which are key brain regions in rMDD [30]. The HPA axis is part of the limbic system. It involves secretion of corticotropin-releasing factor from the hypothalamus, which stimulates secretion of adrenocorticotropic hormone from the pituitary gland; this, in turn, leads to cortisol release from the adrenal glands [101]. Chronic immune dysregulation or inflammation is an important pathologic feature of rMDD, and excessive release of pro-inflammatory cytokines inhibits the negative feedback of the HPA axis, increases the permeability of the blood–brain barrier, reduces serotonin synthesis, disturbs the glutamatergic system, and can even result in depression relapse [102].

**Table 2:** Clinical and neuroimaging findings related to the number of depressive episodes or depression relapse

Study	Characteristics of MDD samples	Number of participants per group		Brain regions	Method
		MDD	HC		
Serra-Blasco et al. (2013) [76]	22 with first-episode, 22 with remitted–recurrent, and 22 with treatment-resistant/chronic MDD	66	32	dmPFC, left insula	Voxel-based morphometry
Treadway et al. (2015) [77]	Medication-free MDD	52	51	Dentate, dmPFC	Volumetric analyses
Stratmann et al. (2014) [78]	35 with first-episode and 97 with rMDD	132	132	Right hippocampus, right amygdala	Voxel-based morphometry
Meng et al. (2014) [79]	25 with rMDD	25	25	Right putamen	Graph-based methods
Greicius et al. (2007) [80]	MDD	28	20	sgACC	Independent component analysis
Jacobs et al. (2016) [81]	17 with active MDD and 34 with remitted MDD	51	26	Left PCC and left inferior frontal gyrus, right middle frontal gyrus, left amygdala with the right anterior insula, caudate and claustrum	Functional connectivity
Lythe et al. (2015) [84]	31 MDD without relapse and 25 MDD with relapse	56	39	Right superior anterior temporal lobe and the sgACC	Functional connectivity
Workman et al. (2016) [85]	30 MDD without relapse and 17 MDD with relapse	47	35	Left sgACC, right sgACC	Functional connectivity
Zaremba et al. (2018) [86]	23 MDD without relapse and 37 MDD with relapse	60	54	Insular and dlPFC	Voxel-based morphometry

Abbreviations: *dlPFC* dorsolateral prefrontal gyrus, *dmPFC* dorsomedial prefrontal gyrus, *HC* healthy controls, *PCC* posterior cingulate cortex, *rMDD* recurrent major depressive disorder, *sgACC* subgenual anterior cingulate cortex

## Conclusions

The literature suggests a strong association between inflammatory processes and rMDD. Inflammation interacts with brain circuits via complicated direct and indirect pathways, including neuronal, immune-mediated, and neuroendocrine-mediated signaling. Remarkably, regions within the DMN, SN, and CEN (which are the “hotspots” involved in MDD and have been identified in numerous imaging studies) are also the regions correlated with high levels of pro-inflammatory cytokines. Subtypes of depression, such as rMDD as discussed here, should be regarded as being associated with specific patterns of inflammation. In this aspect, neuroimaging is at a very early stage and more studies are needed to better understand the relationship between inflammation and neuroimaging markers in rMDD. Further studies are also needed to clarify the mechanism of brain function regulation by inflammation in rMDD.

**Table 3:** Representative MRI studies investigating a putative association between inflammation and structural/functional changes in the brain in MDD

Study	Characteristics of MDD samples	Number of participants per group		Brain regions	Method
		MDD	HC		
Felger et al. (2016) [98]	Medically stable MDD	48	0	Ventral striatum and vmPFC	Functional connectivity
Meier et al. (2016) [99]		73	91	mPFC	Cortical volume, thickness, and surface area
Frodl et al. (2012) [100]	MDD	40	43	Hippocampus	Manual tracing of the bilateral hippocampal and amygdala structure

Abbreviations: *HC* healthy controls, *MDD* major depressive disorder, *mPFC* medial prefrontal gyrus, *vmPFC* ventromedial prefrontal gyrus

## Abbreviations

ACC: Anterior cingulate cortex
CEN: Central executive network
COX-2: Cyclooxygenase-2
CRP: C-reactive protein
dlPFC: Dorsolateral prefrontal cortex
DMN: Default mode network
dmPFC: Dorsomedial prefrontal cortex
fALFF: Fractional amplitude of low-frequency fluctuations
FC: Functional connectivity
fMRI: Functional magnetic resonance imaging
HPA: Hypothalamic−pituitary−adrenal
IDO: Indoleamine 2,3 dioxygenase
IL: Interleukin
MAPK: Mitogen-activated protein kinase
MDD: Major depressive disorder
mPFC: Medial prefrontal cortex
MRI: Magnetic resonance imaging
NE: Norepinephrine
NF-kB: Nuclear factor kappa-light-chain-enhancer of activated B cells
PCC: Posterior cingulate cortex
PET: Positron emission tomography
rMDD: Recurrent major depressive disorder
sgACC: Subgenual anterior cingulate cortex
SN: Salience network
STAT 1a: Signal transducer and activator of transcription 1a
TNF-α: Tumor necrosis factor alpha
TRD: Treatment-resistant depression
vACC: Ventral anterior cingulate cortex
vmPFC: Ventromedial prefrontal cortex
VNS: Vagus nerve stimulation
